# Association between biochemical control and comorbidities in patients with acromegaly: an Italian longitudinal retrospective chart review study

**DOI:** 10.1007/s40618-019-01138-y

**Published:** 2019-11-18

**Authors:** A. Colao, L. F. S. Grasso, M. Di Cera, P. Thompson-Leduc, W. Y. Cheng, H. C. Cheung, M. S. Duh, M. P. Neary, A. M. Pedroncelli, R. Maamari, R. Pivonello

**Affiliations:** 1 Dipartimento di Medicina Clinica e Chirurgia, Sezione di Endocrinologia, Università Federico II di Napoli, Via S. Pansini 5, 80131 Naples, Italy; 2 Dipartimento di Medicina e Scienze della Saluta di V. Tiberio, Università degli Studi del Molise, Campobasso, Italy; 3 Analysis Group, Inc., Boston, MA USA; 4 Novartis Pharmaceuticals Corporation, East Hanover, NJ USA; 5 Novartis Pharma AG, Basel, Switzerland

**Keywords:** Acromegaly, Growth hormone, Insulin-like growth factor I, Comorbidity, Pituitary diseases

## Abstract

**Purpose:**

Achieving biochemical control (normalization of insulin-like growth factor-1 [IGF-1] and growth hormone [GH]) is a key goal in acromegaly management. However, IGF-1 and GH fluctuate over time. The true potential impact of time-varying biochemical control status on comorbidities is unclear and relies on multiple, longitudinal IGF-1 and GH measurements. This study assessed the association between time-varying biochemical control status and onset of selected comorbidities in patients with acromegaly.

**Methods:**

Medical charts of adults with confirmed acromegaly and ≥ 6 months of follow-up at an Italian endocrinology center were reviewed. Patients were followed from the first diagnosis of acromegaly at the center until loss to follow-up, chart abstraction, or death. Biochemical control status was assessed annually and defined as IGF-1 ≤ the upper limit of normal, or GH ≤ 2.5 µg/L in the few cases where IGF-1 was unavailable. Time-varying Cox models were used to assess the association between biochemical control status and comorbidities.

**Results:**

Among 150 patients, 47% were female, average age at diagnosis was 43.1, and mean length of follow-up was 10.4 years. Biochemical control was significantly associated with a lower hazard of diabetes (HR = 0.36, 95% CI 0.15; 0.83) and cardiovascular system disorders (HR = 0.54, 95% CI 0.31; 0.93), and a higher hazard of certain types of arthropathy (HR = 1.68, 95% CI 1.04; 2.71); associations for other comorbidities did not reach statistical significance.

**Conclusion:**

Results further support the importance of achieving biochemical control, as this may reduce the risk of high-burden conditions, including diabetes and cardiovascular system disorders. The association for arthropathy suggests irreversibility of this impairment. Due to limitations, caution is required when interpreting these results.

## Introduction

Acromegaly is a rare chronic disease with a worldwide prevalence of 40–130 per million and an incidence of 3–4 per million [1, 2]. It is characterized by the oversecretion of growth hormone (GH), most commonly due to a pituitary tumor [2, 3]. Elevated GH levels induce the liver to overproduce insulin-like growth factor 1 (IGF-1), leading to a progressive somatic disfigurement and a wide range of systemic manifestations [3]. As a result, patients typically present with acral overgrowth, including exaggerated growth of the hands and feet, facial overgrowth, including prognathism, and soft tissue hypertrophy [3].

Diagnosis of acromegaly is prompted by clinical suspicion of the disease and confirmed with a biochemical evaluation. Elevated serum IGF-1 is the recommended biomarker for diagnosing acromegaly. The diagnosis is confirmed biochemically by the detection of increased IGF-1 concentrations and high GH levels that are not suppressible during an oral glucose tolerance test (OGTT). Elevated levels of IGF-1 and GH are associated with increased mortality [4]. The standard mortality ratio (SMR) for patients with acromegaly with elevated IGF-1 (above the patient’s upper limit of normal) levels is higher than that of the general population (SMR = 2.5, 95% confidence interval [CI] 1.6–4.0), as is the SMR for patients with acromegaly with GH > 2.5 µg/L (SMR = 1.9, 95% CI 1.5–2.4) [4]. However, mortality curves of patients with serum GH < 1 µg/L and normal IGF-1 levels are similar to those of the unaffected individuals [5].

Pituitary surgery represents the primary therapy for most patients, except for patients with a high surgical risk, those who refuse surgery, and those with invasive mostly unresectable tumors. For a majority of patients, however, pharmacotherapy aiming to achieve biochemical control (i.e., normalization of IGF-1 and GH levels) is required as first- or second-line therapy, either because the tumor was only partially resected, or because surgery was not advisable [6, 7].

Biochemical control status (controlled vs. uncontrolled) is defined according to the patient’s levels of IGF-1 and GH, which are known to fluctuate over time for reasons both related and unrelated to acromegaly. With the aforementioned pharmacotherapy that targets biochemical control, IGF-1 and GH may increase or decrease depending on the patient’s response to treatment. Biochemical assessments of IGF-1 and GH are also subject to inter-assay variations based on the reliability of the instruments with which they are measured [8]. Moreover, levels of IGF-1 and GH are subject to influences by other factors, including patient’s diet, sleep and exercise patterns, systemic illness, and concurrent medications [7]. While the majority of patients achieve sustained biochemical control over time when receiving treatment, the pace at which biochemical levels normalize varies for each patient, and a considerable proportion of patients never reach biochemical control [9, 10]. Therefore, relying on IGF-1 and GH measurements from one single point in time may be inaccurate as this may be insufficiently representative of the patient’s true biochemical control status. To obtain a more accurate characterization of the patient’s biochemical control status, multiple measures of IGF-1 and GH over time, via longitudinal monitoring, are needed.

Acromegaly is associated with multiple comorbidities, such as diabetes mellitus, sleep apnea, arthropathy, cardiovascular system disorders (e.g., hypertension), and menstrual irregularities [1]. Despite the key role of biochemical control in the prognosis of acromegaly, the association between biochemical control and the onset of these comorbidities has not been sufficiently investigated [11–15]. From the limited number of studies that have assessed this relationship, data suggest that elevated IGF-1 and/or GH levels at diagnosis may be associated with arthropathy [13], heart disease [11, 15], malignant neoplasms [15], diabetes mellitus [11, 12, 14, 15], hypertension [11, 12], and sleep apnea [11]. However, all such studies relied on IGF-1 and GH measurements at one fixed time point. For reasons described previously, past studies failed to account for the temporal fluctuations of IGF-1 and GH and may have under- or over-stated patients’ biochemical control status over time [16]. It is, therefore, unclear what the true association is between biochemical control, when properly and carefully assessed over the time course, and a range of comorbidities. Given these limited observational data on this important topic, the present study aimed to estimate the association between time-varying biochemical control status and the onset of selected comorbidities associated with acromegalic disease, using retrospective longitudinal data from patient medical charts from an endocrinology center in Italy.

## Methods

### Data source

Data were extracted retrospectively from medical charts of patients diagnosed with acromegaly and treated at the endocrinology center at the University Federico II in Naples, Italy (*the center*). Data were collected via an electronic case report form (CRF) developed by the study authors. This study protocol was reviewed and approved by the University Federico II local ethics committee.

### Patient selection and study design

To be included in this study, patients were required to meet study eligibility criteria, which were having a diagnosis of acromegaly confirmed by a physician, being at least 18 years of age at the time of the first acromegaly diagnosis, having at least two available readings of GH and/or IGF-1 in their medical charts, and having at least 6 months of follow-up at the center.

**Fig. 1:**
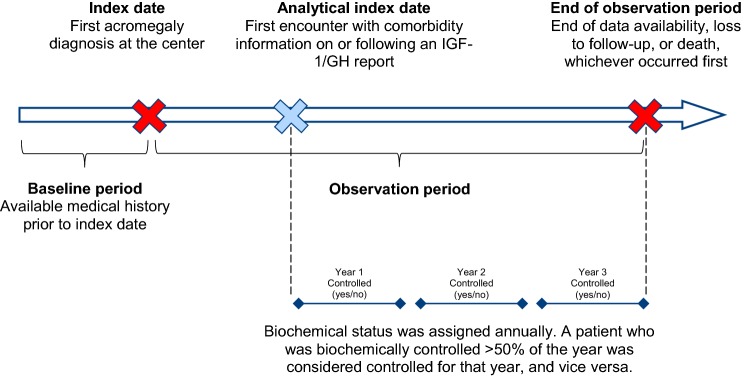
Study design scheme

Figure 1 shows the study design scheme. The study *index date* was defined as the first acromegaly diagnosis at the center. The *baseline period* was defined as medical history prior to and excluding the index date. The *observation period* spanned from the index date to the end of data availability, loss to follow-up, or death, whichever occurred first. All data available in the patients’ medical chart were included, as this was a retrospective, real-world, non-interventional study. As such, no specific procedure was implemented to ensure a regular follow-up or systematic assessment of comorbidities beyond usual care.

### Variables

Biochemical control during the observation period was defined based on IGF-1 levels (i.e., IGF-1 levels less than or equal to a patient’s upper limit of normal). In the absence of IGF-1 levels, random GH less than or equal to 2.5 µg/L was used to define biochemical control. While recent guidelines suggest using a threshold of random GH less than or equal to 1.0 µg/L, random GH less than or equal to 2.5 µg/L was chosen to reflect clinical practice and to account for the fact that some GH measurements were taken on older, less sensitive equipment. Biochemical control was assessed from the first reading of IGF-1 or GH available on or following the index date in the patient’s chart, onwards (thereafter referred to as the *analytical index date*, see Fig. 1). This was re-assessed every time a new IGF-1 or GH measurement was available in the patient’s charts and carried forward daily. Biochemical control status was treated as a dichotomous variable (controlled vs. uncontrolled) and was attributed for every year following the analytical index date. A year during which a patient was biochemically controlled for more than half of the days that year was qualified as “controlled” and vice versa (Fig. 1).

Comorbidities during the baseline period and the observation period assessed in this study included arthropathy, cancer, cardiovascular system disorders (as a composite and also separated into components: hypertension, myocardial hypertrophy, and others), cerebrovascular disease, colon polyps, endocrine and metabolic system disorders (as a composite and also separated into components: dyslipidemia, nodular thyroid disease, glucose metabolism abnormality, gonadal and menstrual disorders, and obesity), and sleep apnea.

Demographic characteristics assessed at the time of the index date included age, sex, and race/ethnicity, and year of the index date. Use of acromegaly-targeting therapies was assessed during the observation period, and included transsphenoidal surgery, somatostatin analogs (SSAs: lanreotide, octreotide, pasireotide), dopamine agonists (cabergoline, bromocriptine), growth hormone receptor antagonists (GHRAs: pegvisomant), and/or radiation therapy.

### Analyses

Demographic characteristics assessed as of the index date and comorbidities during the baseline period were reported using descriptive statistics. Continuous variables were summarized by means, standard deviations (SD) and medians. Categorical variables were summarized by frequencies and proportions. Use of acromegaly-targeting therapies and comorbidities during the observation period were reported using frequencies and proportions.

The association between biochemical control and onset of comorbidities was estimated using a time-varying multivariable Cox regression model. To respect the temporality of comorbidities ensuing from biochemical control status, patients who had a diagnosis of the comorbidity prior to the analytical index date were not entered in the model because they were not at risk of the comorbidity onset. These diagnoses included those recorded upon the collection of medical history as of the index date (baseline period), or prior to the availability of biochemical status (analytical index date). Variables used for model adjustment included age and sex, when applicable (for example, sex was not applicable for sex-specific comorbidities, such as menstrual disorders). Hazard ratios (HRs), 95% CIs, and *p* values were reported. A *p* value of 0.05 was used to define statistical significance.

All analyses were performed using SAS software versions 9.3 and 9.4 (SAS Institute, Cary, NC).

## Results

A hundred and fifty patients met eligibility criteria and were included in the study. The length of the observation period was 10.4 years (SD 7.6). A total of 38 patients (25.3%) had an observation period that lasted 15 years or longer. Four deaths (2.7%) were reported during the observation period. Mean time between assessments of biochemical control was 1.0 years (SD 0.8) while mean time between reporting of comorbidities was 0.9 years (SD 0.5).

Demographic characteristics assessed as of the index date, and comorbidities during the baseline period are presented in Table 1. Patients were, on average, 43.1 years old as of the index date (SD 12.1), 47.3% were female, and all were Caucasian. Most patients (81.3%) had an index date on or after the year 2000.

**Table 1:** Demographic characteristics assessed as of the index date and comorbidities during the baseline period

Number of patients, *N*	150
Age as of the index date, mean ± SD [median]	43.1 ± 12.1 [42.0]
Female, *n* (%)	71 (47.3)
Race, *n* (%)	
Caucasian	150 (100.0)
Year of the index date, *n* (%)	
Before 1990	9 (6.0)
1990–1999	19 (12.7)
2000–2009	70 (46.7)
2010 and after	52 (34.7)
Comorbidities at baseline, *n* (%)	
Arthropathy^a^	33 (22.0)
Hands	6 (4.0)
Spine	4 (2.7)
Hips	3 (2.0)
Knees	3 (2.0)
Other	14 (9.3)
Unknown	6 (4.0)
Cancer	7 (4.7)
Thyroid	3 (2.0)
Breast	1 (0.7)
Choroid plexus	1 (0.7)
Colon	1 (0.7)
Stomach	1 (0.7)
Cardiovascular system disorders	44 (29.3)
Hypertension^b^	33 (22.0)
Myocardial hypertrophy^c^	10 (6.7)
Other^d^	9 (6.0)
Cerebrovascular disease^e,f^	2 (1.3)
Colon polyps^g^	6 (4.0)
Endocrine and metabolic system disorders	64 (42.7)
Nodular thyroid disease^e^	36 (24.0)
Gonadal and menstrual disorders^h^	20 (13.3)
Oligomenorrhea (35 days to 6 months)^i^	8 (11.3)
Amenorrhea (> 6 months)^i^	5 (7.0)
Polymenorrhea (< 21 days)^i^	0 (0.0)
Glucose metabolism abnormality	14 (9.3)
Diabetes mellitus^j^	10 (6.7)
Impaired glucose tolerance^k^	4 (2.7)
Dyslipidemia	12 (8.0)
Hypercholesterolemia^l^	9 (6.0)
Hypertriglyceridemia^m^	2 (1.3)
Obesity^n^	8 (5.3)
Metabolic syndrome^o^	8 (5.3)
Sleep apnea^p^	13 (8.7)

*SD* standard deviation

^a^Confirmed by physical examination or radiographs (Kellgren and Lawrence score ≥ 2). These categories are not mutually exclusive

^b^Systolic blood pressure (SBP) ≥ 140 mmHg, or diastolic blood pressure (DBP) ≥ 90 mmHg

^c^Confirmed by echocardiography or cardiac MRI

^d^Confirmed by blood tests, X-rays, electrocardiogram, Holter monitoring, echocardiogram, cardiac catheterization, CT scan, or MRI scan. These included arrhythmia, atrial fibrillation, heart attack, ischemic cardiopathy, and valvular insufficiency

^e^Confirmed by MRI scan, CT scan, electroencephalogram, serum D-dimer levels, or cardiac evaluation

^f^Cerebrovascular disease included strokes and unspecified brain injuries

^g^Confirmed by colonoscopy

^h^Includes hypogonadism

^i^The proportion of patients with this comorbidity calculated out of 71

^j^Fasting glucose > 126 mg/dL at two consecutive measurements or glucose ≥ 200 mg/dL 2 h after oral glucose tolerance test (OGTT)

^k^Fasting glucose < 126 mg/dL and glucose ≥ 140 and < 200 mg/dL 2 h after OGTT

^l^Total cholesterol levels > 200 mg/dL

^m^Triglycerides levels > 150 mg/dL

^n^Body mass index > 30 kg/m^2^

^o^Metabolic syndrome was defined as having glucose metabolism abnormality and at least two of the following conditions recorded during a 1-year period: diabetes, hypertension, hypertriglyceridemia, and obesity. From World Health Organization. Definition, diagnosis and classification of diabetes mellitus and its complications: report of a WHO consultation. Part 1, Diagnosis and classification of diabetes mellitus. Online. http://apps.who.int/iris/bitstream/10665/66040/1/WHO_NCD_NCS_99.2.pdf. Accessed 13 Oct 2017. Patients were considered assessed for metabolic syndrome if glucose metabolism abnormalities were assessed with at least one of the following: hypertension, dyslipidemia, or obesity

^p^Confirmed by polysomnography or patient-reported symptoms

The three most common comorbidities during the baseline period were endocrine and metabolic system disorders (42.7%), cardiovascular system disorders (29.3%), and arthropathy (22.0%).

Acromegaly-targeting therapy use during the observation period is presented in Table 2. A total of 102 patients (68.0%) underwent transsphenoidal surgery. Radiation therapy was used by six patients (4.0%). The most commonly used SSAs were lanreotide (*n* = 88 [58.7%]) and octreotide (*n* = 81 [54.0%]). SSAs were used as first-line treatment by 86 patients (57.3%), as second-line treatment in 49 patients (32.7%), and as third- or fourth-line treatment in 34 patients (22.7%). Dopamine agonists were used by 51 patients (34.0%), mostly in second, third, or fourth line of treatment. GHRAs were used by 45 patients (30.0%), mostly in third or fourth line.

**Table 2:** Use of acromegaly-related therapies and procedures during the observation period

Number of patients, *N*	150
Transsphenoidal surgery, *n* (%)	102 (68.0)
Phamarcological treatment, *n* (%)	
Somatostatin analogs	128 (85.3)
Line of therapy	
First line	86 (57.3)
Second line	49 (32.7)
Third or fourth line	34 (22.7)
Unknown	4 (2.7)
Medication	
Lanreotide	88 (58.7)
Octreotide	81 (54.0)
Pasireotide	7 (4.7)
Dopamine agonists	51 (34.0)
Line of therapy	
First line	11 (7.3)
Second line	25 (16.7)
Third or fourth line	16 (10.7)
Unknown	0 (0.0)
Medication	
Cabergoline	49 (32.7)
Bromocriptine	2 (1.3)
GH receptor agonists (pegvisomant)	45 (30.0)
Line of therapy	
First line	4 (2.7)
Second line	15 (10.0)
Third or fourth line	26 (17.3)
Unknown	2 (1.3)
Radiation therapy, *n* (%)	6 (4.0)

*GH* growth hormone

The association between biochemical control and the onset of comorbidities is shown in Table 3. Among patients at risk (i.e., those not diagnosed prior to the analytical index date), the most common diagnoses included endocrine and metabolic system disorders (*n* = 66/70 [94.3%]), cardiovascular system disorders (*n* = 59/93 [63.4%]), and arthropathy (*n* = 20/103 [48.5%]).

**Table 3:** Association between biochemical control and comorbidities following the analytical index date

Comorbidity	Incident cases^a^, *n*_cases_/*n*_at risk_ (%)	Hazard ratio^b^ (95% CI)	*p* value^b^
Arthropathy^c^, *n* (%)	50/103 (48.5)	1.07 (0.70; 1.65)	0.746
Spine	34/140 (24.3)	0.97 (0.49; 1.92)	0.929
Hips	25/143 (17.5)	1.08 (0.46; 2.53)	0.856
Hands	8/139 (5.8)	2.05 (0.51; 8.27)	0.315
Knees	8/145 (5.5)	0.71 (0.25; 2.04)	0.530
Other	52/125 (41.6)	1.68 (1.04; 2.71)	0.032*
Unknown	6/143 (4.2)	0.35 (0.05; 2.42)	0.287
Cancer, *n* (%)	10/143 (7.0)	0.42 (0.14; 1.25)	0.119
Cardiovascular system, *n* (%)	59/93 (63.4)	0.54 (0.31; 0.93)	0.027*
Hypertension^d^	47/106 (44.3)	0.67 (0.37; 1.19)	0.172
Myocardial hypertrophy^e^	55/129 (42.6)	0.71 (0.41; 1.22)	0.212
Other^f^	53/137 (38.7)	0.98 (0.59; 1.61)	0.924
Cerebrovascular disease^g,h^, *n* (%)	9/148 (6.1)	0.99 (0.19; 5.30)	0.992
Colon polyps^i^, *n* (%)	22/138 (15.9)	0.52 (0.19; 1.39)	0.190
Endocrine and metabolic system, *n* (%)	66/70 (94.3)	1.01 (0.78; 1.33)	0.917
Dyslipidemia	90/128 (70.3)	1.22 (0.86; 1.74)	0.266
Hypercholesterolemia^j^	87/132 (65.9)	1.17 (0.80; 1.71)	0.415
Hypertriglyceridemia^k^	48/144 (33.3)	1.49 (0.85; 2.64)	0.167
Nodular thyroid disease^g^	61/99 (61.6)	1.08 (0.69; 1.70)	0.729
Glucose metabolism abnormality	53/128 (41.4)	0.64 (0.36; 1.14)	0.134
Diabetes mellitus^l^	31/137 (22.6)	0.36 (0.15; 0.83)	0.017*
Impaired glucose tolerance^m^	31/141 (22.0)	1.32 (0.68; 2.53)	0.409
Gonadal and menstrual disorders^n^	51/121 (42.1)	1.21 (0.70; 2.09)	0.495
Obesity^o^	49/137 (35.8)	0.81 (0.44; 1.51)	0.508
Metabolic syndrome^p^, *n* (%)	36/137 (26.3)	0.53 (0.26; 1.08)	0.082
Sleep apnea^q^, *n* (%)	41/123 (33.3)	0.88 (0.49; 1.58)	0.673

*CI* confidence interval

*Significant at the 5% level

^a^Patients at risk were those who did not have a diagnosis prior to the analytical index date

^b^Hazard ratios and *p* values were estimated using time-varying Cox regression models controlling for age and sex, when applicable. Hazard ratios < 1 mean that biochemical control was negatively associated with the onset of the diagnosis (i.e., protective effect of biochemical control). Only comorbidities with at least five events are reported

^c^Confirmed by physical examination or radiographs (Kellgren and Lawrence score ≥ 2). These categories are not mutually exclusive

^d^Systolic blood pressure (SBP) ≥ 140 mmHg, or diastolic blood pressure (DBP) ≥ 90 mmHg

^e^Confirmed by echocardiography or cardiac MRI

^f^Confirmed by blood tests, X-rays, electrocardiogram, Holter monitoring, echocardiogram, cardiac catheterization, CT scan, or MRI scan. These included arrhythmia, aortic aneurysm, aortic dilation, atrial dilation, atrial enlargement, atrial fibrillation, bradycardia, carotid atherosclerosis, chronic myocardial infarction, extrasystoles, diastolic dysfunction, heart attack, heart failure, history of IMA, history of ischemia, ischemic cardiopathy, mitral insufficiency, mitral valve regurgitation, pulmonary hypertension, pulmonary thromboembolism, right bundle branch block, sclerosis, sinus bradycardia, systolic murmur, tricuspid insufficiency, tricuspid valve regurgitation, valve sclerosis, and valvular insufficiency

^g^Confirmed by MRI scan, CT scan, electroencephalogram, serum D-dimer levels, or cardiac evaluation

^h^Cerebrovascular disease included strokes and unspecified brain injuries

^i^Confirmed by colonoscopy

^j^Total cholesterol levels > 200 mg/dL

^k^Triglyceride levels > 150 mg/dL

^l^Fasting glucose > 126 mg/dL at two consecutive measurements or glucose ≥ 200 mg/dL 2 h after oral glucose tolerance test (OGTT)

^m^Fasting glucose < 126 mg/dL and glucose ≥ 140 and < 200 mg/dL 2 h after OGTT

^n^Includes hypogonadism

^o^Body mass index > 30 kg/m^2^

^p^Metabolic syndrome was defined as having glucose metabolism abnormality and at least two of the following conditions recorded during a 1-year period: diabetes, hypertension, hypertriglyceridemia, and obesity. From World Health Organization. Definition, diagnosis and classification of diabetes mellitus and its complications: report of a WHO consultation. Part 1, Diagnosis and classification of diabetes mellitus. Online. http://apps.who.int/iris/bitstream/10665/66040/1/WHO_NCD_NCS_99.2.pdf. Accessed 13 Oct 2017. Patients were considered assessed for metabolic syndrome if glucose metabolism abnormalities were assessed with at least one of the following: hypertension, dyslipidemia, or obesity

^q^Confirmed by polysomnography or patient-reported symptoms

Biochemical control was statistically significantly associated with a lower hazard of developing diabetes mellitus (HR = 0.36, 95% CI 0.15; 0.83, *p* = 0.017) and cardiovascular system disorders (HR = 0.54, 95% CI 0.31; 0.93, *p* = 0.027).

Biochemical control was also statistically significantly associated with a higher hazard of developing arthropathy (other/unspecified joints only [including diffuse arthropathy], HR = 1.68, 95% CI 1.04; 2.71, *p* = 0.032). Hazard ratios for other comorbidities did not reach statistical significance.

## Discussion

The current study presented data on 150 patients with acromegaly followed for an average of 10 years and demonstrated that biochemical control was statistically significantly associated with delayed onset of diabetes and cardiovascular system disorders. Furthermore, it was associated with the onset of arthropathy of unspecified joints (i.e., other than spine, hips, hands, and knees). Unlike previous research on the topic, this study considered biochemical control status changes longitudinally, which captured patients’ different rates of progress during their disease to either remission or potential disease recurrence. To the best of our knowledge, this is the first study to quantify this association in a time-varying fashion.

Due to the fluctuations of patients’ biochemical control status during the course of their follow-up, biochemistry should be considered as a dynamic factor rather than a “fixed” disease severity indicator (such as elevated IGF-1 at baseline, for example). In an associated analysis using these data, longitudinal data for patients’ biochemical status were observed to follow two distinct trends over the first 10 years of biochemical monitoring: one increasing trend (patients’ probability of being biochemically controlled increased, *n* = 110 [73.3%]) and one stable trend (patients’ probability remained low and stable, *n* = 40 [26.7%]) (unpublished). Moreover, when IGF-1 and GH levels were examined separately, these followed four and three different temporal trends, respectively (unpublished). Such variations cannot be accurately captured by a single measurement of IGF-1 or GH at baseline. In addition, the technique used in this study accounted for the number of years during which the patient was biochemically controlled or uncontrolled prior to the diagnosis of comorbidities. In a 2011 retrospective study, Jayasena and colleagues found that patients with prolonged exposure to elevated IGF-1 and GH levels were more likely to have diagnoses of impaired glucose tolerance, cardiovascular system disorders, and diabetes compared to those with a lesser cumulative biochemical burden [17]. Similarly, a 2012 retrospective study conducted by Varadhan and colleagues reported that cumulative GH exposure was significantly higher in patients with acromegaly who died and in those who had metabolic or vascular events during follow-up [18]. The time-varying Cox regression used in this study accounted for both temporal variations in patients’ biochemical control status, as well as cumulative exposure to elevated IGF-1 and GH levels.

There was a statistically significant association between biochemical control and the onset of cardiovascular system disorders (as a group, HR = 0.42, *p* = 0.027). There was a similar trend for hypertension and myocardial hypertrophy individually, while these did not reach statistical significance (HR = 0.67, *p* = 0.172 and HR = 0.71, *p* = 0.212, respectively). These results further support previous, similar findings [11, 15, 17]. Elevated levels of IGF-1 and GH may lead to cardiomyopathy in three ways: myocyte growth and structure, cardiac contractility, and vascular function [19]. Clinical manifestations include ventricular hypertrophy, diastolic and systolic dysfunction, and valvular regurgitation [19]. Among patients with acromegaly, chronic IGF-1 and GH excess cause changes to cardiac morphology, which is known as “acromegalic cardiomyopathy” [20]. While data on acromegalic cardiomyopathy were not specifically collected in this study, it is likely that this complex condition was reflected in the high incidence of hypertension and myocardial hypertrophy that was observed in this study following the analytical index date. Since some patients may have been diagnosed prior to the index date, it is possible that cardiovascular system disorders due to acromegalic cardiomyopathy could have occurred prior to the analytical index date. This may explain the high prevalence of these disorders observed during the baseline period (*n* = 44 [29.3%]).

There was also a statistically significant association between biochemical control and reduced risk for diabetes (HR = 0.36, *p* = 0.017). With the exception of obesity (HR = 0.81, *p* = 0.508), other endocrine and metabolic system disorders did not seem to show a similar trend (dyslipidemia: HR = 1.22, *p* = 0.266; nodular thyroid disease: HR = 1.08, *p* = 0.729; gonadal and menstrual disorders: HR = 1.21, *p* = 0.495). The association between biochemical control and diabetes has been observed many times in similar, previous research [11, 12, 14, 15]. The pathophysiology of diabetes in patients with acromegaly is well understood and is not the focus of this study [21]. Of note, patients with acromegaly who also have diabetes are more susceptible to develop extra-pituitary neoplasms than non-diabetic patients with acromegaly [22]. Therefore, achieving biochemical control in patients with acromegaly may also reduce the likelihood of extra-pituitary neoplasms through the mediator effect of diabetes, though the association between biochemical control and cancer did not reach statistical significance in this study (HR = 0.42, *p* = 0.119).

The association between biochemical control and arthropathy of other, unspecified joints (i.e., not the spine, hips, hands, or knees) also reached statistical significance (HR = 1.68, *p* = 0.032). This type of arthropathy also included diffuse arthropathy. While the progression of some comorbidities may be slowed or even reversed through biochemical control, bone and cartilage abnormalities may persist because of the irreversibility of the conditions due to long-term exposure to elevated GH [23]. While short-term improvement of arthropathy has been reported among patients shortly after initiating SSAs [24, 25], a prospective study that assessed the course of acromegalic arthropathy patients found that long-term biochemical remission did not stop or reverse the progression of radiological features of acromegalic arthropathy [26]. A similar phenomenon was also observed for vertebral fractures among patients with acromegaly, in spite of long-term biochemical control [27]. Moreover, a 2013 study of 71 patients with acromegaly found that patients with severe arthropathy (*n* = 19) had significantly lower levels of IGF-1 (and higher body mass index) than patients with less severe arthropathy (*n* = 21) [28]. The authors hypothesized that this could be explained by the effect of elevated IGF-1 on osteophytosis with joint space preservation [29]. Finally, there could also be an effect of SSAs on joint structure since SSAs may have an inhibitive effect on cartilage and somatostatin-binding sites that are found on bone cells [26, 30].

## Limitations

This study is subject to a few limitations. First, data were collected at a single endocrinology center in Naples, Italy. Therefore, if a patient was treated or diagnosed with one of the comorbidities under study outside of this specific center (such as during a medical encounter with their general practitioner prior to being referred to the center), this information may be missing from the database. Furthermore, inherently to chart review studies, the quality of the findings rely on consistent and accurate medical charting. Second, as with all observational studies, the impact of unobserved confounders (e.g., diet) could not be assessed. For example, it was not possible to include smoking status and body mass index in the multivariable Cox model since these informations were not collected in CRF. This could have had an impact on the precision of the HR estimated, notably for the hazard of developing cardiovascular system disorders. However, there is no reason to believe that patients have systematically different body mass indexes or smoking habits depending on their biochemical control status, so the conclusions of this study are unlikely to have been meaningfully impacted. Other unobserved factors include time-varying hormonal levels, and family history or genetic predisposition for comorbidities. For example, elevated urotensin II levels were shown to be associated with cardiovascular comorbidities in acromegalic patients [31]. Third, due to the sample size and availability of multiple possible treatment options, it was not possible to adjust for patients’ treatment(s). It is possible that the onset of comorbidities may have been caused or delayed by a patient’s treatment regardless of their biochemical control status, but such an effect could not be captured in the present analysis. Fourth, the relatively low sample size in the current study may have limited our ability to the detect associations for other comorbidities due to the lack of statistical power. Fifth, while reflective of the clinical practice at the time, relying on GH to define the patient’s biochemical control status is not as precise as using IGF-1. However, this was done in very few instances (less than 2% of the time) and, therefore, has likely no material implications on our findings. Sixth, because of the nature of the data source (i.e., partnering with an existing team of physicians who currently treat patients with acromegaly), there may have been a lack of longitudinal data to provide an accurate estimate of mortality. Finally, results may not be generalizable to outside the context of the center in Naples. For example, treatment practice may vary by geographic region, as well as available and publicly reimbursed medications.

## Conclusion

This study quantified the association between biochemical control and the onset of various comorbidities presumed to be associated with acromegalic disease through the use of an observational study. To the best of our knowledge, this is the first study to use a longitudinal review of biochemical control in an observational clinical setting with such extended follow-up time to capture biochemical fluctuations, disease recurrence, and cumulative exposure to GH and IGF-1 excess. Results of this analysis suggest that biochemical control may reduce the risk of certain costly conditions, including diabetes and cardiovascular system disorder.
